# Immunoregulatory Cells and Cytokines Discriminate Disease Activity Score 28-Remission Statuses and Ultrasound Grades in Rheumatoid Arthritis Patients with Non-High Disease Activity

**DOI:** 10.3390/ijms25168694

**Published:** 2024-08-09

**Authors:** Lieh-Bang Liou, Yao-Fan Fang, Ping-Han Tsai, Yen-Fu Chen, Che-Tzu Chang, Chih-Chieh Chen, Wen-Yu Chiang

**Affiliations:** 1 Division of Rheumatology, Allergy, and Immunology, Chang Gung Memorial Hospital at Keelung, Keelung City 204, Taiwan; 2 Division of Rheumatology, Allergy, and Immunology, New Taipei Municipal Tucheng Hospital, New Taipei City 236, Taiwan; 3 Division of Rheumatology, Allergy, and Immunology, Chang Gung Memorial Hospital at Linkou, Taoyuan City 333, Taiwan; 4 School of Medicine, Chang Gung University College of Medicine, Taoyuan City 333, Taiwan

**Keywords:** immunoregulatory cytokines, DAS28 scores, immunoregulatory cells, total ultrasound scores, ultrasound synovitis grades

## Abstract

It is not clear whether immunoregulatory cytokines and cells are associated with Disease Activity Score 28 (DAS28) scores and ultrasound grades/scores. Here, we investigated the relationships between immunoregulatory cytokines or cells and different DAS28 scores or ultrasound grades/scores in patients with rheumatoid arthritis (RA). This study enrolled 50 RA patients (with 147 visits) who had remission/low/moderate DAS28-ESR scores (92% in remission and low disease activity) at baseline. Blood was collected and an ultrasound was performed three times in a year. Percentages of regulatory B cells and T regulatory type 1 cells and M2 macrophage numbers in the blood were examined. Plasma levels of 10 immunoregulatory cytokines IL-4, IL-5, IL-9, IL-10, IL-13, IL-27, IL-35, TGF-β1, sTNF-R1, and sTNF-R2 and monocyte chemotactic protein-1 (MCP-1) were assessed using ELISA assay. The correlations of cytokines and cells with different DAS28 scores and ultrasound grades were investigated, and cytokines and cells were compared between different categories of DAS28 scores and ultrasound grades. Plasma TGF-β1 levels were higher in the DAS28-ESR < 2.6 (remission) subgroup than in the DAS28-ESR ≥ 2.6 (nonremission) subgroup (*p* = 0.037). However, plasma TGF-β1 levels were higher in the high ultrasound grade subgroup than those in the low ultrasound grade subgroup (*p* = 0.007). The number of M2 macrophages was lower in the DAS28-MCP-1 < 2.2 subgroup than in the DAS28-MCP-1 ≥ 2.2 subgroup (*p* = 0.036). The levels of TGF-β1, sTNF-R2, IL-10, and IL-27 were higher in patients with high ultrasound grades than in those with low ultrasound grades. IL-27 was also higher in the nonremission DAS28-ESR subgroup than the remission one (*p* = 0.025). Moreover, sTNF-R1 levels in the 2011 American College of Rheumatology/European League Against Rheumatism (ACR/EULAR) remission subgroup were significantly lower than in the 2011 ACR/EULAR nonremission subgroup (*p* = 0.007). This trend was reflected in that lower sTNF-R1 levels correlated with low DAS28-MCP-1 scores (rho = 0.222, *p* = 0.007). We conclude that high plasma TGF-β1 levels indicate the DAS28-ESR remission (<2.6) subgroup and the high ultrasound grade subgroup. IL-27 probably connects the nonremission DAS28-ESR to high ultrasound grades. Low sTNF-R1 levels probably link low DAS28-MCP-1 scores with the 2011 ACR/EULAR remission subgroup. It suggests that incongruent immuno-inflammatory abnormalities exist between DAS28 scores and ultrasound grades, and are also dissimilar among various DAS28-formula categories. Therefore, this study may provide a basis for further research into individual cytokines and immunoregulatory cells behind each DAS28 formula and ultrasound grades/scores.

## 1. Introduction

Rheumatoid arthritis (RA) affects nearly 1% of the population of Taiwan [1]. RA mainly affects the limb joints and leads to the loss of normal joint function and a reduced ability to perform daily activities. RA can eventually lead to the loss of normal functions of life and working ability [2,3]. Continual high disease activity is associated with progressive joint destruction, and low disease activity or remission indicates low radiographic damage [4,5,6]. Therefore, to obtain favorable outcomes, remission or low disease activity should be achieved [5]. Because a Disease Activity Score 28 (DAS28) remission score (DAS28-ESR) of <2.6 does not indicate disease remission accurately [7], other methods and modifications for identifying remission have been devised [8,9,10,11].

Silent progression of bone erosion was demonstrated to occur in 40% of patients with RA with improved DAS28 and DAS28-C-Reactive Protein (DAS28-CRP) scores, as revealed using MRI [12]; the lowest disease activity (i.e., remission) is the most desirable. Nevertheless, cut-off values for MRI that indicate remission have not yet been established or standardized [12]. Moreover, MRI may not be cost-effective. Whether laboratory biomarkers (e.g., monocyte chemotactic protein-1 [MCP-1]) can replace DAS28-ESR in the original DAS28 formula for defining remission warrants further study [11]. In a previous study [11], we established that DAS28-MCP-1 scores are highly correlated with the DAS28-ESR and DAS28-CRP scores of 0.984 and 0.971, respectively.

We noted that a much higher remission rate was associated with DAS28-MCP-1 than with DAS28-ESR according to two definitions of remission [13,14], and a significantly higher remission rate was associated with DAS28-MCP-1 than with Simplified Disease Activity Index (SDAI) according to the 2005 modified American Rheumatism Association (ARA) remission criteria [15].

Because several definitions for clinical remission have been established since 1981 and the criteria for RA remission based on the DAS28 scores are unsatisfactory [8,9,10,11,13], different disease activity measures must be evaluated against objective laboratory biomarkers and tools, such as cytokines and ultrasound.

High serum levels of the proinflammatory cytokines IL-6, IL-8, IL-17A, IL-17F, IL-18, IL-20, and IL-23 were demonstrated to be unrelated to active synovitis defined by ultrasound [16]. Moreover, levels of the proinflammatory cytokines IFN-γ, TNF-α, IL-2, IL-1β, IL-6, IL-8, and IL-18 do not differ between patients with active and inactive RA. Research also indicates that sTNF-R1 and sTNF-R2 levels are considerably elevated in patients with inactive RA [17]. Although serum IL-21 levels (proinflammatory) are correlated with measures of disease activity, they do not predict remission in patients with RA [18]. Patients with RA in remission (DAS28-ESR < 2.6) have similar serum levels of proinflammatory cytokines to those of patients with clinically active RA (DAS28-ESR > 3.2) [19]. These findings indicate that the use of proinflammatory cytokines is impractical for identifying RA remission defined using the standard DAS28-ESR cut-off score.

In the present study, we investigated whether 10 immunoregulatory cytokines (IL-4, IL-5, IL-9, IL-10, IL-13, IL-27, IL-35, TGF-β1, sTNF-R1, and sTNF-R2) and three immunoregulatory cell types (regulatory B cells [Breg] and T regulatory type 1 [Tr1] cells, and alternately activated macrophages [M2 macrophages]) can accurately indicate remission or very low disease activity as they are defined by various disease activity measures (DAS28-ESR < 2.6, DAS28-MCP-1 < 2.2, DAS28-CRP < 2.5, SDAI ≤ 3.3) [15]. In addition, the degree of ultrasound-defined synovitis was used to test the validity of the definition of remission according to these disease activity measures. The interrelationships among immunoregulatory cytokines, immunoregulatory cells, various disease activity measures, and ultrasound-defined synovitis were studied to establish the biological basis of RA remission and assess the superiority of the disease activity measures.

## 2. Results

The demographic and laboratory data, medications, and comorbidities of the 50 patients with RA are displayed in Table 1. There were 38 females and 12 males. The number of 50 was determined as described in Section 4 and Section 4.1. Clinical and laboratory data were collected for 147 visits. The disease duration was 119.8 ± 61.7 months with a range of 10–233 months. Only one patient was diagnosed less than 12 months prior to enrollment.

### 2.1. Comparison and Correlation of Laboratory Data and Ultrasound Grades across Different DAS28-Based Remission Statuses

The percentages of low ultrasound grades (combined grade 0 and 1 synovitis) in the DAS28-CRP < 2.5 (46/61 = 75.4%), SDAI ≤ 3.3 (61/89 = 68.5%), and DAS28-MCP-1 < 2.2 (54/79 = 68.4%) groups (all belong to the remission subgroups) were compared with that in the DAS28-ESR < 2.6 group (65/98 = 66.3%) by means of odds ratios; no difference was identified. The number of RA patients with specific DAS28 score categories at each time point was displayed in Supplementary Table S1.

The percentages of immunoregulatory Breg and Tr1 cells were not different between the DAS28-ESR < 2.6 (remission) and ≥2.6 (nonremission) groups (Table 2). Similarly, no difference was identified between the DAS28-CRP < 2.5, SDAI ≤ 3.3, and DAS28-MCP-1 < 2.2 remission groups and their individual nonremission groups. However, the number of M2 macrophages could be used to significantly discriminate DAS28-MCP-1 < 2.2 from ≥2.2 (the latter had a higher number), but not for comparisons of other DAS28-based statuses (Table 2). Detailed medium and 25–75% quartiles of all data were given in Supplementary Table S2.

**Figure 1:**
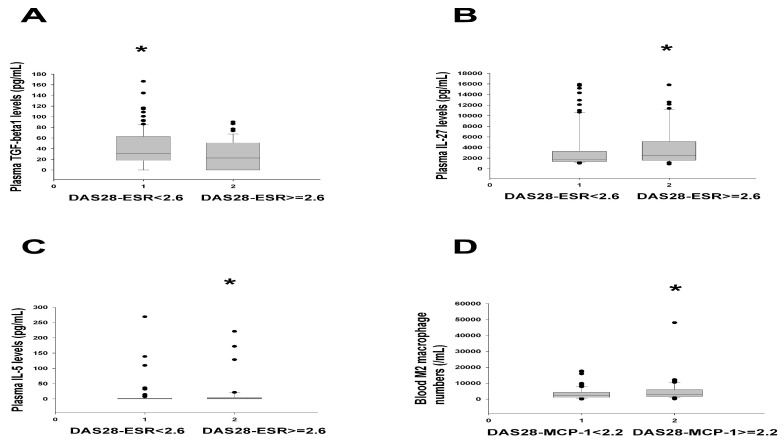
Different cytokine levels or M2 macrophage numbers across DAS28-based remission statuses: (**A**–**C**) Plasma TGF-β1, IL-27, and IL-5 levels significantly differentiated DAS28-ESR < 2.6 (n = 98) from DAS28-ESR ≥ 2.6 (n = 49, having higher values for IL-27 and IL-5) at *p* = 0.037, 0.025, and 0.028, respectively. (**D**) The number of M2 macrophages in the blood discriminated DAS28-MCP-1 < 2.2 (n = 79) from DAS28-MCP-1 ≥ 2.2 (n = 68, having higher M2 macrophage numbers) at *p* = 0.036. All comparisons were conducted using the Mann–Whitney U test. * Asterisks indicate the subgroup had significantly higher cytokine levels or M2 macrophage numbers than another subgroup.

Because no correlation between immunoregulatory cells or cytokines and RA disease activity DAS28 scores has been reported in the literature, we further assessed this association. Among the investigated cytokines, only sTNF-R1 was significantly and positively correlated with DAS28-MCP-1, and the correlation coefficient was low (Table 3). In addition, plasma TGF-β1 levels were higher in the DAS28-ESR < 2.6 subgroup than in the DAS28-ESR ≥ 2.6 subgroup (Figure 1A). However, plasma IL-27 and IL-5 levels were higher in the DAS28-ESR ≥ 2.6 subgroup than in the DAS28-ESR < 2.6 subgroup (Figure 1B,C). In line with the results presented in Table 2, the blood M2 macrophage numbers in the DAS28-MCP-1 ≥ 2.2 subgroup were significantly higher than those in the DAS28-MCP-1 < 2.2 subgroup (Figure 1D). Nevertheless, the percentage of immune-regulatory cells or cytokines in the normal range or higher in remission statuses of different DAS28-score-formulas were not different from each other, except for higher IL-4 levels in SDAI ≤ 3.3 than three other DAS28 scores (Supplementary Table S3).

We further compared cytokine levels between RA patients and healthy controls (Table 4). It demonstrated that plasma IL-9, IL-27, and IL-35 levels in RA patients were significantly higher than those in healthy controls (Table 4). The mean levels of IL-4, IL-5, IL-10, and IL-13 in RA patients were higher than those in healthy controls. However, the mean levels of TGF-β1, sTNF-R1, and sTNF-R2 in RA patients were lower than those in healthy controls (Table 4). Moreover, immunoregulatory cells and cytokines were not different between Anti-CCP-positive and -negative subgroups in RA patients (Supplementary Table S4).

### 2.2. Correlation of Laboratory Data with Total Ultrasound Scores (the Sum of Ultrasound Grades for Grayscale and Power Doppler from All Examined Joints)

**Figure 2:**
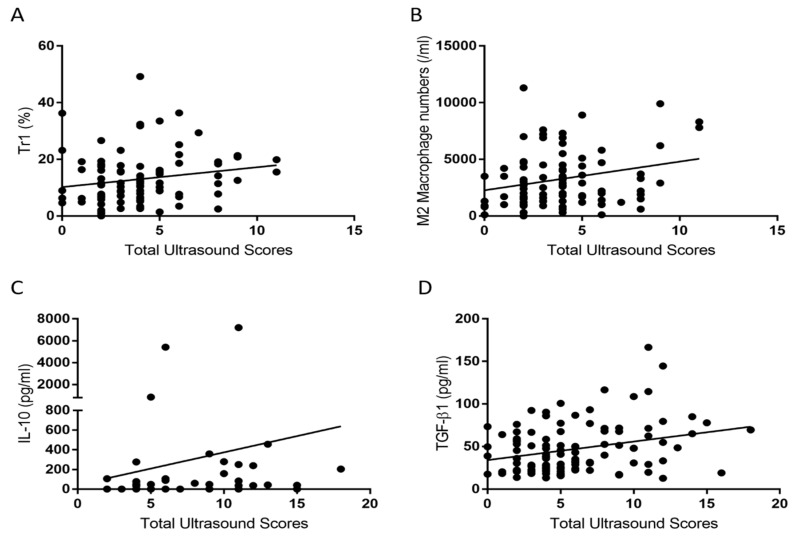
Correlation of cytokines and cells with Total Ultrasound Scores. Ultrasound grades were scored as in ref. [20]: (**A**) The percentage of Tr1 cells (n = 89) correlated with Total Ultrasound Scores (the sum of ultrasound grades for grayscale and power Doppler of all examined joints) in the ultrasound grade subgroup (≤1) at rho = 0.242 and *p* = 0.022. Tr1 is presented as percentages (%). (**B**) The M2 macrophage numbers (n = 89) correlated with Total Ultrasound Scores in the ultrasound grade subgroup (≤1) at rho = 0.221 and *p* = 0.038. (**C**) The IL-10 level (n = 58) correlated with Total Ultrasound Scores in the high ultrasound grade subgroup (>1) at rho = 0.311 and *p* = 0.017. (**D**) The TGF-β1 level (n = 147) correlated with Total Ultrasound Scores at rho = 0.203 and *p* = 0.033. Correlation performed by Spearman’s correlation.

This study revealed that TGF-β1 levels were significantly and positively correlated with Total Ultrasound Scores (the sum of ultrasound grades for grayscale and power Doppler of all examined joints), although the correlation coefficient was low (Table 5 and Figure 2D). Ultrasound grades were assessed by employing the EULAR-OMERACT scoring system [20]. Tr1 cells and M2 macrophages positively correlated with Total Ultrasound Scores in the low ultrasound grade subgroup (≤1) (Table 6 and Figure 2A,B). In contrast, IL-10 positively correlated with Total Ultrasound Scores in the high ultrasound grade subgroup (>1) (Table 6 and Figure 2C). Plasma IL-5 levels revealed an inverse relationship with Total Ultrasound Scores (Table 6).

In contrast, a comparison of the percentage/number of immunoregulatory cells and cytokine levels between low and high ultrasound grades (the latter comprising grade 2 and grade 3) revealed no significant differences (Supplementary Table S5).

### 2.3. Comparisons of Cells and Cytokines between Patients with Low Ultrasound Grades and High Ultrasound Grades for Individual Joints

**Figure 3:**
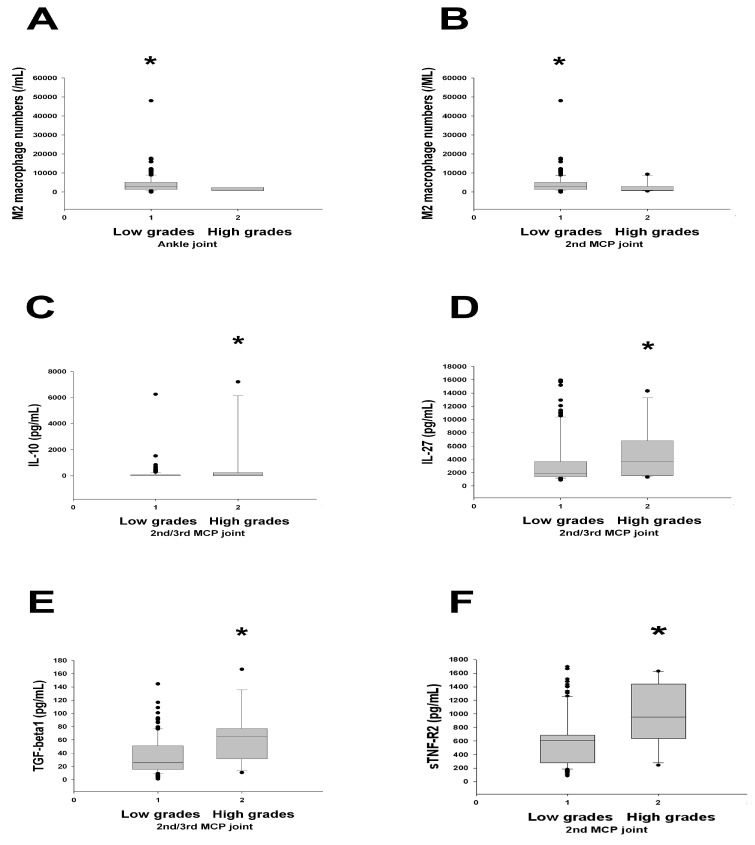
Comparison of M2 macrophage numbers and immunoregulatory cytokines between two groups with ultrasound grades for individual joints. Ultrasound grades were scored as described in a previous study [20]: (**A**) The number of M2 macrophages in the group with low ultrasound grades (grades 0 and 1) (n = 140) was higher than that in the group with high ultrasound grades (grades 2 and 3) (n = 7) for the ankle joint (*p* = 0.038). (**B**) The number of M2 macrophages in the group with low ultrasound grades (n = 137) was higher than that in the group with high ultrasound grades (n = 10) for the 2nd metacarpo-phalangeal (MCP) joint (*p* = 0.027). (**C**) IL-10 levels in the group with high ultrasound grades (n = 15) were higher than those in the group with low ultrasound grades (n = 132) in the second MCP joint (*p* = 0.038). (**D**) IL-27 levels in the group with high ultrasound grades (n = 15) were higher than those in the group with low ultrasound grades (n = 132) in the second and third MCP joint (*p* = 0.002). (**E**) TGF-β1 levels in the group with high ultrasound grades (n = 15) were higher than those in the group with low ultrasound grades (n = 132) for the second and third MCP joints (*p* = 0.007). (**F**) sTNF-R2 levels in the group with high ultrasound grades (n = 10) were higher than those in the group with low ultrasound grades (n = 137) in the second MCP joint (*p* = 0.010). All comparisons were conducted using the Mann–Whitney U test. * Asterisks indicate the subgroup had significantly higher cytokine levels or M2 macrophage numbers than another subgroup.

Some articles correlated disease activity scores and ultrasound findings of individual joints [21,22], which were also examined in our study. We categorized the patients into groups on the basis of whether they had low ultrasound grades (grades 0 and 1) or high ultrasound grades (grades 2 and 3) at all visits. It was noted that the number of M2 macrophages in the patients with low ultrasound grades was higher than those in the patients with high ultrasound grades for the ankle joint (Figure 3A). A similar trend was noted for M2 macrophages in the second metacarpo-phalangeal (MCP) joint (Figure 3B).

In contrast, IL-10 and IL-27 levels were higher in the group with high ultrasound grades than in the group with low ultrasound grades for the second and third MCP joints (Figure 3C and Figure 3D, respectively). That is, increased levels of IL-10 and IL-27, which are immunoregulatory, exist with high ultrasound grades.

Similarly, TGF-β1 and sTNF-R2 levels were higher in the patients with high ultrasound grades than in those with low ultrasound grades for the second and third MCP joints and for the second MCP joint, respectively (Figure 3E and Figure 3F, respectively). The results of TGF-β1 (Figure 3E) were compatible with TGF-β1’s positive correlation with ultrasound grades in Table 5 and Figure 2A. Increased Tr1 percentages in Figure 2 and increased levels of IL-10, IL-27, TGF-β1, and sTNF-R2 here were displayed in patients with high ultrasound grades; these results demonstrate that higher levels of some immunoregulatory cytokines and Tr1 cells are compatible with high ultrasound grades.

### 2.4. Changes in Different Laboratory Data and Disease Activity Measures with Total Ultrasound Scores across Three TIME Points in the 12-Month Period

When we separated all related data into Month 0 (baseline), Month 6, and Month 12, several interesting findings were noted (Table 7). Total Ultrasound Scores gradually decreased from Month 0 to Month 6 and further down to Month 12 (Table 7). This trend was paralleled by the changes in the CRP levels, percentages of regulatory B cells, and plasma levels of IL-4, IL-9, IL-13, IL-35, and sTNF-R2 (Table 7). In contrast, both of the above two presented a reverse trend with ESR, DAS28-ESR, DAS28-CRP, SDAI, DAS28-MCP-1, tender joint count (TJC), and swollen joint count (SJC) (Table 7). Moreover, the number of M2 Macrophages and HAQ-DI scores demonstrated the highest in Month 0, followed by that in Month 12, and the lowest in Month 6 (Table 7). The same was seen for IL-5, IL-10, IL-27, TGF-β1, and sTNF-R1 (Table 7).

### 2.5. Assessments of Immunoregulatory Cytokines and Cells Based on the 2005 Modified ARA Remission Criteria and the 2011 ACR/European League Against Rheumatism (ACR/EULAR) Criteria for Remission

Among 10 cytokines and 3 cells examined, we noted that only the M2 macrophage numbers differed between the fulfillment subgroup of 2005 modified ARA remission criteria [13] (lower in number) and the subgroup not fulfilled (higher in number) (Table 8). This trend was compatible with lower M2 macrophage numbers in DAS28-MCP-1 < 2.2 (remission status) (Table 2 and Figure 1D), but not with those for higher M2 macrophage numbers in the low ultrasound grade subgroup (the ankle joint) (Figure 3A) and in the low ultrasound grade subgroup (the 2nd MCP joint) (Figure 3B). Hence, a contradiction existed between the different correlations mentioned above.

Moreover, sTNF-R1 levels in the 2011 ACR/EULAR remission [14] subgroup were significantly lower than in the 2011 ACR/EULAR nonremission subgroup (Table 9). This trend was reflected in that lower sTNF-R1 levels correlated with low DAS28-MCP-1 scores (Table 3). Hence, lower sTNF-R1 levels probably connected low DAS28-MCP-1 scores with the 2011 ACR/EULAR remission subgroup.

IL-10 levels in the 2011 ACR/EULAR remission subgroup were also significantly lower than in the 2011 ACR/EULAR nonremission subgroup (Table 9). The IL-10 level correlated with Total Ultrasound Scores in the high ultrasound grade subgroup (>1) (Figure 2C and Table 6). However, no corresponding DAS28 remission status or low ultrasound grades was established. Interestingly, IL-5 levels in the 2011 ACR/EULAR remission subgroup were significantly lower than in the 2011 ACR/EULAR nonremission subgroup (Table 9); it is compatible with the lower IL-5 levels in DAS28-ESR < 2.6 (remission status) (Figure 1C).

Altogether, the trend of change over time in sTNF-R1, IL-10, and IL-5 levels over M0, M6, and M12 were compatible with the trend of change over time in M2 macrophage numbers over M0, M6, and M12 (Table 7).

## 3. Discussion

Studies have reported that proinflammatory cytokines neither correlate with ultrasound grades nor are able to discriminate inactive from active RA [16,17,19]. Therefore, we investigated whether immunoregulatory cytokines and cells are correlated with different DAS28-based remission statuses and with ultrasound grades/scores.

Similar to a previous study [23], we demonstrated that plasma IL-27 levels were higher in individuals with DAS28-ESR ≥ 2.6 than in those with DAS28-ESR < 2.6 (Figure 1B). Although proinflammatory IL-6 levels are high in the active RA synovitis, immunoregulatory IL-27 (an IL-6-related cytokine) levels in the RA synovium match with IL-6 levels [24]. Mouse studies have obtained similar findings [25,26]. Elevated IL-27 levels in the inflamed synovium likely counteract continuing adaptive immune responses; this is supported by showing higher IL-27 levels in the groups with both DAS28-ESR ≥ 2.6 group (Figure 1B) and higher ultrasound grades (Figure 3D).

In addition, in the present study, plasma IL-5 levels were higher in the DAS28-ESR ≥ 2.6 group than in the DAS28-ESR < 2.6 group (Figure 1C), which has not been reported by other studies. Higher plasma IL-5 levels were correlated inversely with low Total Ultrasound Scores in the ultrasound grade ≤ 1 group (Table 6). Whether this result is due to the incompatibility of DAS28-ESR scores with ultrasound grades warrants further study.

In this study, plasma TGF-β1 levels were higher in the DAS28-ESR < 2.6 subgroup than in the DAS28-ESR ≥ 2.6 subgroup (Figure 1A), which was in contrast to the finding that higher plasma TGF-β1 levels were positively correlated with higher ultrasound grades (Figure 2D). One article published in 2019 showed that “serum TGF-β1 was significantly lower in group A (DAS28 score ≥ 5.0) than that in group B (3.2 < DAS28 score < 5.0) (*p* < 0.001)” [27]. That is, RA patients with lower (moderate) DAS28-ESR scores have higher TGF-β levels than RA patients with high DAS28-ESR scores, which is analogous to what we obtained (Figure 1A). Moreover, another report illustrated that the “average serum level of TGF-β1 was positively associated with Kellgren-Lawrence grades” of osteoarthritis [28]. That is, higher serum TGF-β1 levels are correlated with the severity of osteoarthritis, which is similar to our results in Figure 2D for RA patients. Such discrepancy may be related to the contrasting role of TGF-β1 in OA patients and healthy controls [29]. We formulated the following hypothesis for the current study: Elevated levels of TGF-β1 in the DAS28-ESR remission subgroup probably represent an anti-inflammatory reaction in the RA process. In particular, the DAS28-ESR remission subgroup has low percentages that fulfill the 2005 modified ARA remission criteria (36.99%) and the 2011 ACR/EULAR remission definition (49.13%) (see Table 3, ref. [15]). Hence, the DAS28-ESR remission subgroup may contain a significant portion of RA patients having high inflammation, similar to those seen in the high ultrasound score subgroup (correlating with elevated levels of TGF-β1, Figure 2D). Nevertheless, no report has ever been published on the correlation between serum TGF-β1 levels and musculoskeletal ultrasound as in Figure 2D in RA patients. Therefore, only a future study on this aspect can elucidate such discrepancy between disease activity measures and ultrasound grades in terms of plasma TGF-β1 correlation.

Although no correlation was identified between T regulatory cells (FoxP3 + T cells) and grayscale synovial proliferation and power Doppler signals [30], we found a moderate positive correlation between ultrasound grades and the percentage of Tr1 cells (Figure 2A). In addition, the DAS28-MCP-1 ≥ 2.2 group had higher blood M2 macrophage numbers than the DAS28-MCP-1 < 2.2 group did (Table 2, Figure 1D); these findings reflected a phenomenon: higher percentages or numbers of immunoregulatory cells (Tr1 and M2 macrophages) corresponded to higher ultrasound grades or higher DAS28-MCP-1 scores (Figure 2A and Figure 1D). However, this finding did not occur in the DAS28-ESR, DAS28-CRP, and SDAI scores.

The number of CD206^+^M2-like macrophages was correlated inversely with the DAS28-ESR scores [31]. That is, the RA patients with lower numbers of M2-like macrophages had higher disease activity (DA28-ESR). However, our study revealed that RA patients with the nonremission status (DAS28-MCP-1 ≥ 2.2) had higher numbers of blood M2 macrophages (Figure 1D, Table 2). The reason for this discrepancy in the results is unclear. However, more than half of the RA patients in the previous study had DAS28-ESR scores of >4, whereas in the current study, all RA patients had DAS28-ESR scores of <4 (Table 1). In addition, we stained M2 macrophages with CD14^+^CD80^-^CD163^+^ CD206^+^, which is different from those (i.e., CD163^+^ CD206^+^) used as M2-like macrophages in the previous study [31]. This may be another factor explaining the discrepancy.

The number of M2 macrophages could be used to differentiate low and high ultrasound grades for joints of large and small sizes (Figure 3A: ankle joints; Figure 3B: 2nd MCP joints). Cytokine levels could be used to discriminate low and high ultrasound grades for mostly small joints (MCP joints in Figure 3C–F). Whether such differences regarding the joints involved in RA have any pathophysiological implications requires further study. Lastly, the results in Figure 3F (higher sTNF-R2 levels positively related to higher ultrasound grades) were similar in trend to that in Table 7 (sTNF-R2 over a 12-month period positively parallelled to Total Ultrasound Scores). This corresponding trend probably indirectly suggests that our use of Total Ultrasound Scores is at least partly compatible with the ultrasound grades scoring system as described by the EULAR-OMERACT ultrasound task force [20].

It was interesting to note that, in Table 7, DAS28-ESR, DAS28-CRP, SDAI, and DAS28-MCP-1 scores gradually increased from Month 0 to Month 6 and ultimately to the highest in Month 12, similar to the ESR levels. In contrast, this trend was contrasted by the decreasing trend of Breg cells and plasma levels of IL-4, IL-9, IL-13, IL-35, and sTNF-R2 over the course of 12 months (Table 7). Thus, two patterns of similar and contrasted trends to change over time probably indicated partly common underlying immunoregulatory cell and cytokine mechanisms in DAS28-ESR, DAS28-CRP, SDAI, and DAS28-MCP-1 scores. Consequently, the next interesting question is whether these different-formula scores were highly correlated as reported in ref. [11] was further investigated (Supplementary Table S6). The correlation of DAS28-MCP-1 scores with DAS28-ESR and DAS28-CRP scores is similar to those in ref. [11], though the correlation coefficients were lower in the current study. This is probably because most RA patients (92%) in the current study were in remission and had low disease activity. Moreover, it showed that the correlation coefficient between DAS28-MCP-1 and DAS28-ESR scores was higher with the ESR ≥ 28 mm/hr subgroup. This trend is similar to those presented in Table 2 of ref. [15]; however, the CRP ≥ 10 mg/L subgroup, having only 13 visits, possibly gave inconsistent results in the current study.

## 4. Materials and Methods

### 4.1. Participants and Study Design

The study protocol was approved by the Institutional Review Board of Chang Gung Memorial Hospital. After they provided written informed consent, 50 patients with RA who fulfilled the 1987 American College of Rheumatology (ACR) criteria for RA and had a DAS28-ESR score belonging to remission/low/moderate disease activity categories (an arbitrary selection, due to ESR data being unknown on the day of collecting those RA patients and the first ultrasound examination was only performed on the day of enrollment). Nevertheless, DAS28-ESR scores for most of the enrolled RA patients (80%) were less than 2.6 (that is, in remission) were recruited as candidates (with 147 visits in total). This was compatible with our initial rationale to include mainly patients in remission (DAS28-ESR < 2.6, n = 40 and 80%) and with low disease activity (2.6 ≤ DAS28-ESR ≤ 3.2, n = 6 and 12%). There were only 4 patients enrolled having 3.2 < DAS28-ESR ≤ 5.1 (moderate in disease activity), that is, 8% of the enrolled patients; such patients constituted 12.2% (n = 6) of the Month 6 patient number and 18.8% (n = 9) of the Month 12 patient number. The number of 50 patients with RA was determined by analogy with the patient numbers in 2 articles on studying ultrasound remission and DAS28-ESR-based remission (with 55 and 52 RA patients, respectively) [16.19]. Patients with RA who were aged between 20 and 80 years were randomly enrolled through consecutive recruitment from our rheumatology outpatient departments. They underwent assessments (described in the subsequent paragraph), including blood collection, clinical assessment, and ultrasound examination every 6 months for a period of 12 months (3 visits per patient).

Our primary objective was to compare the biologic basis (immunoregulatory cytokines and cells) for the differences in the remission rates defined as DAS28-ESR < 2.6, DAS28-MCP-1 < 2.2, DAS28-CRP < 2.5, and SDAI ≤ 3.3 [15]. Similar assessments were also performed for the 2005 modified ARA criteria and the 2011 ACR/EULAR criteria for remission [13,14]. From each patient, 20 mL of blood was collected and tested for cytokines, immunoregulatory Breg and Tr1 cells, and M2 macrophages to explore whether a similar immunoregulatory mechanism underlies remission as defined by different disease activity measures (i.e., DAS28-ESR, DAS28-MCP-1, DAS28-CRP, and SDAI). Our secondary objective was to compare the percentages of immunoregulatory cells and ultrasound grades for synovitis in patients in remission or nonremission, as defined using the cut-off points of various disease activity measures [15].

### 4.2. Clinical Assessment of Patients at 147 Visits

At each visit, we collected data on current medications, comorbidities, Health Assessment Questionnaire-Disability Index (HAQ-DI) items [32,33], morning stiffness, TJC, SJC, and patients’ and evaluators’ global assessments (PGA and EGA, respectively) of disease activity (visual analog scale [VAS; in cm]), ESR, and CRP.

### 4.3. Analysis of Immunoregulatory Cytokines and Cells

Initially, blood from collection tubes containing ethylenediaminetetraacetic acid was centrifuged at 650× *g* (Kubota 2420, Tokyo, Japan) and at room temperature (25 °C) for 10 min into layers of plasma and cells. After the plasma was removed, cells were blended with an equal volume of phosphate-buffered saline. The cell solution was then assembled into 50 mL plastic tubes containing 10 mL of Ficoll–Paque (Cytiva Europe, Uppsala, Sweden), and further centrifuged at 800× *g* and at 25 °C for 30 min to obtain interface cells as peripheral blood mononuclear cells (PBMCs) as described [34].

The collected plasma was examined for the following cytokines: IL-4, IL-5, IL-9, IL-10, IL-13, IL-27, IL-35, TGF-β1, sTNF-R1, and sTNF-R2 [17,35,36]. Similarly, plasma from 40 healthy controls was analyzed for a part of these cytokines by following a previously described procedure [17]. The results were used to compare the RA and healthy control groups.

Ten plasma immunoregulatory cytokines and MCP-1 were analyzed as per the manufacturer’s recommendations for cytokine assay (R&D Systems, Minneapolis, MN, USA). Rheumatoid factor (RF) was measured through nephelometry by using the N Latex RF Kit (Siemens Healthcare Diagnostics Products GmbH, Marburg, Germany) and anti-cyclic citrullinated peptide (anti-CCP) antibodies were examined by ELISA assay (Quanta FLASH CCP3 IgG ELISA kit; Inova Diagnostics, San Diego, CA, USA) twice at baseline and Month 12.

With PBMCs, Breg and Tr1 cells and M2 macrophages were stained using the following antibodies and analyzed by using the BD FACSCalibur System: Breg: CD19^+^CD5^+^CD1d^hi^ (anti-CD19-fluorescein isothiocyanate [FITC], anti-CD5-phycoerythrin [PE], and anti-CD1d^hi^-allophycocyanin [APC]); Tr1: CD4^+^CD49b^+^CD223^+^ [anti-CD4-FITC, anti-CD49b-AF647(APC), and anti-LAG-3-PE (CD223-PE)]; and M2 macrophages: CD14^+^CD80^-^CD163^+^CD206^+^ (anti-CD14-FITC, CD80-PE Cy7, CD163-PE, and CD206-APC). All staining reagents were obtained from BD biosciences (Franklin Lakes, NJ, USA). The number of cells collected for analysis was at least 20,000. The isotype IgG and process of staining for Breg and Tr1 cells and M2 macrophages are given below. One example of a staining procedure with images was displayed in Supplementary Figure S1.

[Breg]: 1st step gating: Mouse IgG1k-FITC as an isotype control and a background staining of lymphocytes to gate/obtain CD19-FITC-positive B cells; and 2nd step gating: The double staining of mouse IgG1k-PE and IgG1k-APC as isotype controls and background staining for CD19^+^ B cells to gate/obtain CD5^+^-PE and CD1d^high^-APC Breg cells.

[Tr1]: 1st step gating: Mouse IgG1k-FITC as an isotype control and a background staining of lymphocytes to gate/obtain CD4-FITC-positive T cells; and 2nd step gating: The double staining of mouse IgG1k-PE and IgG2a-AF647 as isotype controls and background staining for CD4^+^ T cells to gate/obtain CD223-PE (LAG-3-PE) and CD49b-AF647 double-positive T cells.

[M2 macrophages]: 1st step gating: mouse IgG2a-FITC as an isotype control and a background staining of monocytes to gate/obtain CD14-FITC-positive monocytes; and 2nd step gating: The triple staining of mouse IgG1k-PE, IgG1k-PE Cy7, and IgG1k-APC as isotype controls and background staining for CD14^+^ monocytes to gate firstly CD206^+^-APC cells (containing both M1 and M2 macrophages) and then gate/obtain CD163^+^-PE (containing both M1 and M2 macrophages), and lastly CD80^—^PE Cy7 M2 macrophages (M1 macrophages are CD80^+^).

The percentages of Breg and Tr1 cells were calculated by their proportions based on CD19^+^ B cells and CD4^+^ T cells. The M2 macrophage number/mL was obtained with the final cell number shown on the closing Flow data sheet, by which the final cell number times the reciprocal of its solution volume (0.1 mL).

### 4.4. Calculation of DAS28 Scores

The DAS28 scores were calculated as previously described by using the following equations: DAS28-ESR score = [0.56 × √TJC] + [0.28 × √SJC] + 0.70 × ln[ESR] + 0.014 PGA (in mm), DAS28-CRP score = ([0.56 × √TJC] + [0.28 × √SJC] + (0.36 × ln [CRP; in mg/L]) + 1) + (0.014 × PGA [in mm]) + 0.96), and SDAI = (SJC + TJC + PGA [VAS; in cm] + EGA [VAS; in cm] + CRP [in mg/dL]) [37]. DAS28-MCP-1 scores were calculated using the following modified DAS28 formula: DAS28-MCP-1 = 0.56 × √TJC + 0.28 × √SJC + 0.39 × ln(MCP-1) + 0.014 × (PGA [in mm]) [11]. The cut-off points of DAS28-MCP-1 for the remission, low, moderate, and high disease activity categories were <2.2, ≤3.6, and ≤4.8, respectively (analyzed from the same data set [15]) [also stated in ref. [38]].

### 4.5. Ultrasound Examination of Patients with RA at 147 Visits

The ultrasound measurements were conducted by three rheumatologists who have had thorough experience in musculoskeletal ultrasound techniques. To maintain measurement uniformity, three rheumatologists, who rotated in performing assessments, had an earlier consensus meeting to set out standardized procedures for ultrasound scoring [38]. The bilateral second and third metacarpophalangeal (MCP), wrist, elbow, shoulder, and knee joints of the patients were analyzed using grayscale and power Doppler and the combination of both by employing the EULAR-OMERACT scoring system [20] at baseline (Month 0), Month 6, and Month 12. Moreover, Total Ultrasound Scores were termed by us as the sum of grayscale plus power Doppler from all examined joints [38]. Ultrasound examination was performed using Acuson P300 (Siemens, Münich, Germany) with the ultrasonic probe LA435 and a frequency of 6 to 18 MHz. This approach is pioneering because plasma immunoregulatory cytokines have not ever been employed to correlate with the degree of ultrasound-defined synovitis and with different disease activity measures of RA. Two examples of ultrasound grading are shown in Supplementary Figure S2.

### 4.6. Statistical Analysis

The percentages of patients with DAS28 < 2.6, DAS28-CRP < 2.5, DAS28-MCP-1 < 2.2, or SDAI ≤ 3.3 (all belong to the remission groups) with cytokine levels and the percentages of immunoregulatory cells in these remission groups defined on the basis of DAS28 scores were compared using the Mann–Whitney U test for nonnormal data. The visits in the DAS28-CRP < 2.5, DAS28-MCP-1 < 2.2, and SDAI ≤ 3.3 groups [15] with ultrasound-defined minimal (grade 1) or no (grade 0) synovitis [20] were compared with those in the DAS28 < 2.6 group by calculating the odds ratio. In addition, the results of 3 visits were assessed together. Because only 3 visits (2%) were missed out of the scheduled 150, no correction was made for the missed visits. This study also analyzed the correlations between immunoregulatory cytokines or immunoregulatory cells and ultrasound-defined no or minimal synovitis to obtain insights into RA inflammation. Accordingly, we compared the cells and cytokines between patients with low ultrasound grades (grades 0 and 1) and high ultrasound grades (grades 2 and 3). A *p* value of less than 0.05 was considered to indicate statistical significance. All statistical analyses were performed through SPSS 22.0 version (IBM, SPSS Inc., Chicago, IL, USA).

## 5. Conclusions

In summary, we provide results here from the first comprehensive study that comprises 10 immunoregulatory cytokines, three immunoregulatory cell types, four different DAS28-related scores, and ultrasound findings, as shown above. Notably, the number of M2 macrophages significantly discriminated DAS28-MCP-1 < 2.2 (lower number in remission) from DAS28-MCP-1 ≥ 2.2 (nonremission) (Table 2), and sTNF-R1 was positively correlated with DAS28-MCP-1 (Table 3); thus, both sTNF-R1 and M2 macrophages are good indicators of DAS28-MCP-1-based remission status. In contrast, the number of M2 macrophages was higher in the group with low ultrasound grades than in that with high ultrasound grades (Figure 3A,B). Hence, further studies of macrophages [39] in the role of DAS28-MCP-1’s participation in indicating 2005 modified ARA remission and 2011 ACR/EULAR remission definitions [15] will be very informative, considering that the number of M2 macrophages could discriminate the DAS28-MCP-1 remission status from the nonremission status (Table 2) and low/high ultrasound grades (Figure 3A,B), and sTNF-R1 correlated with DAS28-MCP-1 scores (Table 3). It could especially elucidate the importance and mechanism of employing DAS28-MCP-1 in future clinical practice. We also afford opportunities to study other parameters, including IL-5, IL-27, TGF-β1, and Tr1 levels, in relation to DAS28-related remissions and ultrasound changes in future studies.

Moreover, we pioneer in demonstrating that both paralleled changes in Total Ultrasound Scores and CRP levels, percentages of Breg cells, and plasma levels of IL-4, IL-9, IL-13, IL-35, and sTNF-R2 over a 12-month period contrasted inversely with ESR, DAS28-ESR, DAS28-CRP, SDAI, DAS28-MCP-1, TJC, and SJC (Table 7).

When we compared immunoregulatory cytokines and cells based on the 2005 modified ARA remission/nonremission and the 2011 ACR/EULAR criteria for remission/nonremission, it is suggested that lower M2 macrophage numbers, the DAS28-MCP-1 < 2.2 remission status, and the 2005 modified ARA remission status were probably inter-related among each other. This is obviously reflected in that the DAS28-MCP-1 < 2.2 remission status is superior to DAS28-ESR in indicating the above two remission definitions in Parts (A) and (B) of Table 10. In particular, DAS28-MCP-1 < 2.2 remission is better than SDAI remission in indicating the 2005 modified ARA remission (Part B, Table 10) as similarly previously described [15].

Most importantly, results from this study indicate that dissimilar immuno-inflammatory abnormalities exist between DAS28 scores and ultrasound grades/scores, and are also different among various DAS28-formula categories. Nevertheless, we probably provide a basis for further research into individual cytokines and immunoregulatory cells behind each DAS28 formula and ultrasound grade/score.

## Figures and Tables

**Table 1:** Demographic and laboratory data, medications, and comorbidities of patients with RA at baseline (Month 0).

Categories	Items	Mean ± S.D.	Range
Demographic data			
	Gender	F:M = 38:12	
	Total visits	147	
	Age	56.0 ± 11.5	27–73
	Disease duration (months)	119.8 ± 61.7	10–233
Laboratory and clinical data			
	CRP (mg/L)	3.88 ± 4.24	0.2–16.7
	ESR (mm/hr)	13.9 ± 8.8	2–39
	RF (IU/mL)	73.8 ± 117.0	0.9–512
	Anti-CCP (CU)	463.4 ± 631.6	4.2–2776.7
	DAS28-ESR	2.08 ± 0.72	0.49–3.90
	TJC	0.5 ± 1.0	0–4
	SJC	0.2 ± 0.5	0–2
	HAQ-DI score	0.2 ± 0.5	0–2
Corticosteroids			
	Prednisolone (n = 2, 4.0%)	2.5 ± 0.0 mg/day	2.5–2.5
	Methylprednisolone (n = 1, 2.0%)	4.0 mg/day	-
cDMARDs			
	Sulfasalzine (n = 35, 70.0%)	2057.1 ± 627.5 mg/day	500–3000
	Methotrexate (n = 30, 60.0%)	10.7 ± 4.10 mg/week	5–20
	Hydroxychloroquine (n = 25, 50.0%)	316.0 ± 98.7 mg/day	200–400
	Leflunomide (n = 8, 16.0%)	17.5 ± 4.6 mg/day	10–20
	Azathioprine (n = 6, 12.0%)	100.0 ± 31.6 mg/day	50–150
bDMARDs			
	Abatacept (n = 4, 8.0%)	395.9 ± 157.7 mg/4 weeks	166.7–500.0
	Golimumab (n = 1, 2.0%)	50 mg/4 weeks	-
	Certolizumab (n = 1, 2.0%)	400 mg/4 weeks	-
tDMARDs			
	Tofacitinib (n = 1, 2.0%)	10 mg/day	-
Comorbidities			
	Hypertension (n = 23, 46%)		
	Type 2 diabetes mellitus (n = 1, 2%)		
	Chronic kidney disease (n = 4, 8%)		
	Myocardial infarction (n = 1, 2%)		

These parameters were measured upon patient enrollment. CRP: C-reactive protein; ESR: erythrocyte sedimentation rate; RF: rheumatoid factor; anti-CCP: anti-cyclic citrullinated peptide; DAS28-ESR: Disease Activity Score 28-ESR; TJC: tender-joint count; SJC: swollen-joint count; HAQ-DI: Health Assessment Questionnaire-Disability Index. cDMARDs: conventional disease-modifying anti-rheumatic drugs; bDMARDs: biologic disease-modifying anti-rheumatic drugs; tDMARDs: targeted disease-modifying anti-rheumatic drugs. Normal ranges: CRP < 5 mg/L, ESR < 30 mm/h for women and ESR < 20 mm/h for men, RF < 15 IU/mL, and anti-CCP ≤ 20 CU.

**Table 2:** Comparison of the percentage or number of immunoregulatory cells in remission versus nonremission for different DAS28-score-based remission statuses.

Remission Statuses	Breg	Tr1	M2 Macrophages
DAS28-ESR < 2.6 (n = 98, remission) vs. ≥2.6 (n = 49, nonremission)	0.341	0.698	0.871
DAS28-CRP < 2.5 (n = 62, remission) vs. ≥2.5 (n = 84, nonremission)	0.503	0.983	0.983
SDAI ≤ 3.3 (n = 90, remission) vs. >3.3 (n = 56, nonremission)	0.461	0.374	0.342
DAS28-MCP-1 < 2.2 (n = 79, remission) vs. ≥2.2 (n = 68, nonremission)	0.542	0.269	0.036 *

DAS28: Disease Activity Score 28; SDAI: Simplified Disease Activity Index. *p* values are presented. Breg (immunoregulatory B cells) and Tr1 (T regulatory type 1 cells) were compared in terms of their percentages (%), and M2 macrophages were compared in terms of the number per milliliter (number/mL). The numbers behind the DAS28 scores denote the visit numbers. The comparison of M2 macrophage numbers between the DAS28-MCP-1 < 2.2 (n = 79 visits, median = 2000, 25% and 75% percentiles = 1000 and 4400) and DAS28-MCP-1 ≥ 2.2 (n = 68 visits, median = 3100, 25% and 75% percentiles = 1600 and 5675) groups through Mann–Whitney U test provided * *p* = 0.036. Detailed medium and 25–75% ranges of all data are given in Supplementary Table S2.

**Table 3:** Correlation of immunoregulatory cells and cytokines with different rheumatoid arthritis disease activity scores.

Items		DAS28-ESR	DAS28-CRP	SDAI	DAS28-MCP-1
Breg	rho	−0.043	−0.034	−0.075	−0.117
	*p*	0.605	0.681	0.366	0.158
Tr1	rho	−0.047	−0.014	−0.024	−0.094
	*p*	0.577	0.867	0.771	0.258
M2	rho	0.112	0.097	0.089	0.144
macrophages	*p*	0.178	0.246	0.283	0.081
IL-4	rho	0.103	0.123	0.126	0.139
	*p*	0.215	0.139	0.130	0.094
IL-5	rho	0.169	0.137	0.154	0.135
	*p*	0.042	0.099	0.063	0.104
IL-9	rho	0.097	0.100	0.055	0.098
	*p*	0.245	0.230	0.509	0.237
IL-10	rho	−0.060	−0.040	−0.047	−0.137
	*p*	0.471	0.628	0.572	0.099
IL-13	rho	0.125	0.127	0.113	0.059
	*p*	0.133	0.127	0.173	0.477
IL-27	rho	0.092	0.084	0.063	0.114
	*p*	0.269	0.313	0.451	0.167
IL-35	rho	0.005	0.190	0.174	0.120
	*p*	0.951	0.021	0.036	0.147
TGF-β1	rho	−0.154	−0.116	−0.084	−0.141
	*p*	0.064	0.163	0.313	0.088
sTNF-R1	rho	0.115	0.138	0.188	0.222 *
	*p*	0.166	0.970	0.023	0.007 *
sTNF-R2	rho	−0.099	−0.059	−0.025	−0.116
	*p*	0.236	0.483	0.762	0.160

Breg: immunoregulatory B cells; Tr1: T regulatory type 1 cells; M2: alternatively activated macrophage. All cytokines are expressed as pg/mL. Correlation performed by Spearman’s correlation. * Significant at *p*-value < 0.01.

**Table 4:** Comparisons of immunoregulatory cytokines between rheumatoid arthritis (RA) patients and healthy controls.

Type of Cytokines	Healthy Controls (n = 40) (Mean ± S.D. and Ranges or Mean and Ranges)	RA Patients (n = 50) (Mean ± S.D. and Ranges)	*p*-Values
IL-9	42.57 ± 59.09 pg/mL (0–194.16)	55.32 ± 97.37 pg/mL (0–446.44)	0.025
IL-27	3259.65 ± 2386.71 pg/mL (975.16–8704.21)	* 3692.88 ± 3725.70 pg/mL (880.75–15,929.04)	0.034
IL-35	83.51 ± 115.71 pg/mL (0–328.75)	* 869.33 ± 1472.40 pg/mL (0–7426.85)	0.009
IL-4	<31.1 pg/mL	33.56 ± 72.40 pg/mL (0–427.32)	
IL-5	<3.9 pg/mL	11.02 ± 42.24 pg/mL (0–268.92)	
IL-10	<7.8 pg/mL	252.37 ± 1011.63 pg/mL (0–7204.82)	
IL-13	0 (non-detectable)	604.86 ± 1004.64 pg/mL (102.01–6992.33)	
TGF-β1	1165 pg/mL (903–1654)	67.49 ± 29.71 pg/mL (17.52–166.55)	
sTNF-R1	914 pg/mL (484–1407)	516.02 ± 258.30 pg/mL (49.67–1336.79)	
sTNF-R2	1500 pg/mL (829–2262)	972.47 ± 426.35 pg/mL (109.76–1697.60)	

All cytokine data were examined in human plasma. IL-9, IL-27, and IL-35 in healthy controls were examined in this study. Other cytokine data in healthy controls (IL-4, IL-5, IL-10, IL-13, TGF-β1, sTNF-R1, and sTNF-R2) were taken from the manufacturer’s data sheets of R&D Systems, Minneapolis, MN, USA. * IL-27 and IL-35 data in RA patients were calculated from 147 visits. Comparisons were performed by Mann–Whitney U test.

**Table 5:** Correlation between immunoregulatory cells or cytokines and Total Ultrasound Scores.

Items	Rho
Breg	0.151
Tr1	0.166
M2 macrophage	0.063
IL-4	0.059
IL-5	−0.158
IL-9	−0.012
IL-10	0.082
IL-13	0.104
IL-27	0.025
IL-35	−0.09
TGF-β1	0.203 *
sTNF-R1	0.053
sTNF-R2	−0.073

Total Ultrasound Scores: the sum of ultrasound grades [20] for grayscale and power Doppler of all examined joints. Breg: immunoregulatory B cells; Tr1: T regulatory type 1 cells; M2: alternatively activated macrophages, M2 macrophages. All cytokines are expressed as pg/mL. Correlation (n = 147) was performed by Spearman’s correlation. * Significant at *p*-value < 0.05, but all other correlations gave *p*-values > 0.05.

**Table 6:** Correlation between immunoregulatory cells or cytokines and Total Ultrasound Scores in the ultrasound grade subgroups ^#^.

Items	Grade ≤ 1 (n = 89)			Grade > 1 (n = 58)		
	Percentage	rho	*p*-Value	Percentage	rho	*p*-Value
Breg	58.2%	0.044	0.684	41.8%	0.207	0.118
Tr1	59.9%	0.242*	0.022 *	40.1%	−0.046	0.732
M2	61.7%	0.221	0.038 *	38.3%	−0.155	0.246
IL-4	59.4%	−0.026	0.809	40.6%	0.167	0.209
IL-5	50.8%	−0.297	0.005 *	49.2%	−0.197	0.138
IL-9	60.5%	−0.034	0.755	39.5%	0.036	0.787
IL-10	58.3%	−0.057	0.595	41.7%	0.311	0.017 *
IL-13	60.5%	0.074	0.492	39.5%	0.144	0.282
IL-27	60.5%	−0.051	0.638	39.5%	0.017	0.901
IL-35	60.5%	−0.08	0.454	39.5%	−0.23	0.082
TGF-β1	58.6%	0.045	0.721	41.4%	0.286	0.071
sTNF-R1	60.5%	0.011	0.921	39.5%	0.106	0.427
sTNF-R2	60.5%	−0.206	0.053	39.5%	0.067	0.618

^#^ Ultrasound scoring system: see ref. [20]. Combined ultrasound groups: Grade ≤ 1, comprising grade 0 and grade 1; grade > 1, comprising grade 2 and grade 3. Total Ultrasound Scores: the sum of ultrasound grades for grayscale and power Doppler of all examined joints. Breg: immunoregulatory B cells; Tr1: T regulatory type 1 cells; M2: alternatively activated macrophages, M2 macrophages. All cytokines are expressed as pg/mL. Correlation was determined using Spearman’s correlation; * indicates statistical difference with *p*-values < 0.05.

**Table 7:** Changes in different laboratory data and disease activity measures with Total Ultrasound Scores across three time points in the 12-month period.

	M0 (n = 50)	M6 (n = 49)	M12 (n = 48)
CRP(mg/L)	3.88 ± 4.24 (0.2–16.7)	3.71 ± 6.05 (0.2–35.6)	3.63 ± 6.09 (0.2–36.61)
ESR(mm/hr)	13.86 ± 8.83 (2–39)	15.88 ± 14.99 (2–74)	16.45 ± 13.36 (3–65)
RF(IU/mL)	73.97 ± 116.93 (8.38–512)		56.16 ± 104.21 (5–456)
Anti-CCP(CU)	463.42 ± 631.56 (4.19–2776.7)		396.22 ± 525.75 (4.6–1974)
TJC	0.54 ± 0.99 (0–4)	0.96 ± 1.27 (0–6)	1.15 ± 1.44 (0–6)
SJC	0.22 ± 0.46 (0–2)	0.45 ± 0.98 (0–4)	0.46 ± 0.80 (0–4)
DAS28-ESR	2.08 ± 0.72 (0.47–3.90)	2.29 ± 0.75 (0.97–4.11)	2.53 ± 0.92 (0.97–5.08)
DAS28-CRP	2.60 ± 0.65 (1.38–4.08)	2.77 ± 0.78 (1.38–4.42)	2.93 ± 0.94 (1.38–5.52)
SDAI	2.35 ± 3.36 (0.02–15.11)	3.58 ± 4.27 (0.02–17.14)	4.85 ± 5.76 (0.02–20.84)
DAS28-MCP-1	2.03 ± 0.65 (1.40–3.85)	2.36 ± 0.73 (1.48–4.33)	2.61 ± 0.77 (1.58–4.37)
HAQ-DI	0.22 ± 0.46 (0–1)	0.09 ± 0.32 (0–2)	0.13 ± 0.30 (0–1.333)
Total Ultrasound Scores ^#^	6.62 ± 4.38 (0–18)	5.63 ± 3.33 (1–17)	5.35 ± 3.32 (0–16)
Breg cells (%)	55.93 ± 11.92 (20.9–82.9)	54.49 ± 12.22 (17.8–81.3)	46.02 ± 15.04 (0.15–77.2)
Tr1 cells (%)	12.70 ± 8.56 (0.00756–36.3)	13.91 ± 8.83 (2.61–52.5)	13.54 ± 10.58 (1.31–49.2)
M2 Macrophages (/mL)	4838.00 ± 7748.97 (100–48,000)	3467.35 ± 2524.91 (0–11,000)	3547.92 ± 2848.52 (0–12,000)
IL-4 (pg/mL)	33.56 ± 72.40 (0–427.32)	23.05 ± 58.12 (0–327.69)	21.20 ± 47.70 (0–298.40)
IL-5 (pg/mL)	11.02 ± 42.24 (0–268.92)	7.56 ± 28.97 (0–172.03)	9.18 ± 36.68 (0–220.96)
IL-9 (pg/mL)	55.32 ± 96.37 (0–446.44)	52.85 ± 71.22 (0–379.22)	50.09 ± 74.30 (0–376.99)
IL-10 (pg/mL)	252.37 ± 1011.63 (0–7204.82)	172.63 ± 779.45 (0–5411.55)	177.78 ± 926.73 (0–6251.72)
IL-13 (pg/mL)	604.86 ± 1004.64 (102.01–6992.33)	451.60 ± 673.03 (99.56–4572.34)	415.63 ± 770.28 (92.20–5269.50)
IL-27 (pg/mL)	4240.43 ± 4456.56 (1037.52–15,929.04)	3296.35 ± 3286.28 (1072.67–12,168.10)	3527.30 ± 3297.42 (880.75–12,914.61)
IL-35 (pg/mL)	957.05 ± 1768.78 (0–7426.85)	929.91 ± 1443.46 (29.12–5921.65)	716.11 ± 1144.05 (13.64–5091.10)
TGF-β1 (pg/mL)	67.49 ± 29.71 (17.52–166.55)	20.99 ± 15.84 (1.06–77.12)	24.77 ± 13.88 (5.08–58.53)
sTNF-R1 (pg/mL)	516.02 ± 258.30 (49.67–1336.79)	462.02 ± 153.89 (153.35–880.48)	480.21 ± 142.67 (183.20–820.89)
sTNF-R2 (pg/mL)	972.47 ± 426.35 (109.76–1697.60)	599.00 ± 105.35 (293.94–726.07)	298.04 ± 161.95 (83.06–667.55)

^#^ The sum of ultrasound grades for grayscale and power Doppler scores of all examined joints. TJC: tender joint count; SJC: swollen joint count; DAS28: Disease Activity Score 28; SDAI: Simplified Disease Activity Index; HAQ-DI: Health Assessment Questionnaire-Disability Index; Breg: immunoregulatory B cells; Tr1: T regulatory type 1 cells; M2 macrophages: alternatively activated macrophages.

**Table 8:** Comparison of immunoregulatory cytokines and cells in remission versus nonremission statuses for the 2005 modified ARA remission criteria ^#^.

Items	Remission (n = 79 Visits)		Nonremission (n = 68 Visits)	
	Medium or Mean ± S.D.	Quartiles 25–75% or Ranges	Medium or Mean ± S.D.	Quartiles 25–75% or Ranges
Breg cells (%)	52.5 ± 13.0	19.8–81.3	51.9 ± 14.4	0.15–82.9
Tr1 cells (%)	11.7	6.6–18.4	11.2	6.9–17.5
*M2 Macrophages (/mL)	1850.0	1000.0–4250.0	3200.0	1600.0–5800.0
IL-4 (pg/mL)	15.4	6.8–78.6	13.5	1.6–90.1
IL-5 (pg/mL)	1818.7	1499.7–5932.5	2127.8	1300.9–3961.6
IL-9 (pg/mL)	173.3	64.4–1127.7	220.9	84.5–844.3
IL-10 (pg/mL)	1.3	0.0–29.4	0.4	0.0–12.3
IL-13 (pg/mL)	0.6	0.0–2.0	0.4	0.0–2.2
IL-27 (pg/mL)	0.0	0.0–103.8	0.0	0.0–59.6
IL-35 (pg/mL)	241.4	192.7–464.0	224.0	170.6–510.5
TGF-β1 (pg/mL)	29.2	18.2–67.1	26.8	14.1–50.3
sTNF-R1 (pg/mL)	482.9 ± 212.9	49.67–1336.79	489.2 ± 175.4	67.11–1028.09
sTNF-R2 (pg/mL)	650.0	363.4–862.1	588.4	276.4–690.4

^#^ Ref. [13]. Breg: immunoregulatory B cells; Tr1: T regulatory type 1 cells; M2 macrophages: alternatively activated macrophages. All comparisons of cytokines or cells between remission versus nonremission statuses gave *p*-values > 0.05, except for *M2 Macrophages yielding *p* = 0.021 by Mann–Whitney U test.

**Table 9:** Comparison of immunoregulatory cytokines and cells in remission versus nonremission statuses for the 2011 ACR/EULAR remission definition ^#^.

Items	Remission (n = 89 Visits)		Nonremission (n = 58 Visits)	
	Medium or Mean ± S.D.	Quartiles 25–75% or Ranges	Medium or Mean ± S.D.	Quartiles 25–75% or Ranges
Breg cells (%)	52.6 ± 13.1	19.8–82.9	51.6 ± 14.8	0.15–77.00
Tr1 cells (%)	11.7	7.2–28.4	10.7	6.2–17.5
M2 Macrophages (/mL)	2700.0	1250.0–5200.0	2500.0	1250.0–4725.0
IL-4 (pg/mL)	12.4	3.0–33.7	18.9	4.2–119.9
IL-5* (pg/mL)	1720.6	1329.1–3247.7	2415.5	1512.6–6755.1
IL-9 (pg/mL)	171.0	67.6–1081.1	233.8	89.4–858.6
IL-10* (pg/mL)	0.0	0.0–10.7	4.4	0.0–55.7
IL-13 (pg/mL)	0.4	0.0–19.1	0.6	0.0–78.3
IL-27 (pg/mL)	0.0	0.0–59.6	0.0	0.0–78.3
IL-35 (pg/mL)	228.6	168.9–403.1	260.8	176.2–729.0
TGF-β1 (pg/mL)	29.0	18.1–59.5	27.5	13.3–51.2
sTNF-R1* (pg/mL)	451.6 ± 165.5	49.67–880.48	539.5 ± 219.8	158.73–1336.79
sTNF-R2 (pg/mL)	602.2	271.2–685.3	640.9	284.9–696.9

^#^ Ref. [14]. Breg: immunoregulatory B cells; Tr1: T regulatory type 1 cells; M2 macrophages: alternatively activated macrophages. All comparisons of cytokines or cells between remission and nonremission statuses gave *p*-values > 0.05, except for IL-5* and IL-10* yielding *p* = 0.020 and 0.041, respectively, by Mann–Whitney U test. Comparison of sTNF-R1* between remission and nonremission produced *p* = 0.007 by *t*-test.

**Table 10:** Remission rates by two remission definitions in different Disease Activity Score 28 (DAS28) score-based statuses.

	% of Remission (Remission/Nonremission Visits)	Odds Ratio ^#^		% of Remission (Remission/Nonremission Visits)	Odds Ratio ^#^
(A) * The combination of the current study with that published in 2023 (n = 461 visits)					
Fulfillment of 2005 modified ARA remission			Fulfillment of 2011 ACR/EULAR remission		
DAS28-ESR < 2.6	55.17% (96/78)	Reference	DAS28-ESR < 2.6	64.74% (112/61)	Reference
DAS28-CRP < 2.5	74.53% (79/27)	2.803	DAS28-CRP < 2.5	83.97% (89/17)	3.184
SDAI ≤ 3.3	67.63% (94/45)	1.697	SDAI ≤ 3.3	85.61% (119/20)	3.241
DAS28-MCP-1 < 2.2	71.64% (96/38)	2.053	DAS28-MCP-1 < 2.2	75.37% (101/33)	1.667
(B) ^&^ United previous one (n = 835 visits) and (A) (n = 461 visits)					
Fulfillment of 2005 modified ARA remission			Fulfillment of 2011 ACR/EULAR remission		
DAS28-ESR < 2.6	46.11% (160/187)	Reference	DAS28-ESR < 2.6	56.94% (197/149)	Reference
DAS28-CRP < 2.5	72.73% (128/48)	3.419	DAS28-CRP < 2.5	83.52% (147/29)	4.191
SDAI ≤ 3.3	61.30% (160/101)	1.851	SDAI ≤ 3.3	83.91% (219/42)	3.944
DAS28-MCP-1 < 2.2	73.15% (158/58)	3.184	DAS28-MCP-1 < 2.2	77.78% (168/48)	2.647

* (A) The current study containing mainly DAS28-ESR scores ≤ 3.2 (92% on enrollment) was joined with the study published in 2023 ref. [34] containing all >3.2 (100% on enrollment) to encompass all visits as a group to cover whole ranges of rheumatoid arthritis disease activity in terms of DAS28-ESR scores. All odds ratios ^#^ vs. the reference in each category gave *p* < 0.05 in Part (A). Inside parentheses are visit numbers. ^&^ (B) The data in 2020 ref. [15] were mixed with (A) to calculate data shown in (B). All odds ratios ^#^ vs. the reference in each category provided *p* < 0.001 in Part (B). Both DAS28-MCP-1 < 2.2 and DAS28-CRP < 2.5 vs. SDAI ≤ 3.3 for fulfillment of 2005 modified ARA remission offered *p* = 0.006 and 0.013, respectively.

## Data Availability

The data that support the findings of this study are displayed in the article. Others are available from the corresponding author upon reasonable request.

## Supplementary Materials

The following supporting information can be downloaded at: https://www.mdpi.com/article/10.3390/ijms25168694/s1.
