# Supplementary material for: Immunoregulatory Cells and Cytokines Discriminate Disease Activity Score 28-Remission Statuses and Ultrasound Grades in Rheumatoid Arthritis Patients with Non-High Disease Activity
Source: Int J Mol Sci. 2024 Aug 9;25(16):8694. doi: 10.3390/ijms25168694 (PMC11354682; doi:10.3390/ijms25168694)
Supplement: Supplementary file 1 [file ijms-25-08694-s001.zip › ijms-3095788-supplementary.pdf]

**[Supplementary Tables and Figures]**

**Immunoregulatory cells and cytokines discriminate Disease Activity Score 28-remission statuses and ultrasound grades in rheumatoid arthritis patients with non-high disease activity**

Lieh-Bang Liou , Yao-Fan Fang, Ping-Han Tsai , Yen-Fu Chen, Che-Tzu Chang, Chih-Chieh Chen, Wen-Yu Chiang

**Supplementay Table S1.** The number of RA patients in two categories of DAS28 scores at different time points

| | **Month 0 (n = 50)** | **Month 6 (n = 49)** | **Month12 (n = 48)** |
|---|---|---|---|
| DAS28-ESR | | | |
| < 2.6 | 40 | 32 | 27 |
| ≧2.6 | 10 | 17 | 21 |
| DAS28-CRP | | | |
| <2.5 | 21 | 22 | 20 |
| ≧2.5 | 29 | 27 | 27 |
| SDAI | | | |
| ≦3.3 | 36 | 28 | 27 |
| >3.3 | 14 | 21 | 20 |
| DAS28-MCP-1 | | | |
| <2.2 | 36 | 24 | 20 |
| ≧2.2 | 14 | 25 | 28 |

DAS28: Disease Activity Score; SDAI: Simplified Disease Activity Index.

**Supplementary Table S2.** The percentage or number of immunoregulatory cells in remission and nonremission of different DAS28-score-based statuses

| Items | Remission Medium (or Mean± S.D.) | 25% (or the lowest) | 75% (or the highest) | Nonremission Medium (or Mean± S.D.) | 25% (or the lowest) | 75% (or the highest) |
|---|---|---|---|---|---|---|
| Breg (%) | | | | | | |
| DAS28-ESR | (53.10% ± 12.2%) | (19.80%) | (82.90%) | (50.40% ± 16.40%) | (0.15%) | (77.20%) |
| DAS28-CRP | (52.20% ± 14.1%) | (19.80%) | (82.90%) | (52.20% ± 13.60%) | (0.15%) | (77.00%) |
| SDAI | (53.50% ± 13.1%) | (19.80%) | (82.90%) | (50.20% ± 14.60%) | (0.15%) | (77.00%) |
| DAS28-MCP-1 | (53.70% ± 12.7%) | (19.80%) | (82.90%) | (50.40% ± 14.70%) | (0.15%) | (81.30%) |
| Tr1 (%) | | | | | | |
| DAS28-ESR | 11.60% | 7.16% | 17.50% | 11.10% | 6.12% | 18.85% |
| DAS28-CRP | 11.40% | 6.93% | 17.85% | 11.40% | 6.57% | 17.93% |
| SDAI | 11.70% | 6.93% | 18% | 10.80% | 6.54% | 17.80% |
| DAS28-MCP-1 | 12.40% | 7.17% | 17.80% | 11.05% | 6.01% | 18.10% |
| M2 macrophages (number/mL) | | | | | | |
| DAS28-ESR | 2600 | 1000 | 5025 | 2600 | 1500 | 4900 |
| DAS28-CRP | 2900 | 1250 | 4800 | 2500 | 1275 | 5100 |
| SDAI | 2200 | 1150 | 4800 | 2900 | 1525 | 5125 |
| DAS28-MCP-1 | 2000 | 1000 | 4400 | 3100 | 1600 | 5675 |

Breg: B regulatory cells; Trl: T regulatory type 1 cells; shown are medium and 25%-75% range of all data. Remission and nonremission are defined as: DAS28-ESR<2.6 (remission) and ≧2.6 (nonremission); DAS28-CRP<2.5 (remission) and ≧2.5 (nonremission); Simplified Disease Activity Index (SDAI)≦3.3 (remission) and >3.3 (nonremission); DAS28-MCP-1<2.2 (remission) and ≧2.2 (nonremission).

**Supplementary Table S3.** The percentage of immune-regulatory cells or cytokines in normal range or higher in remission statuses of different DAS28-score-formulas

| Items | DAS28-ESR<2.6 (n = 99) | DAS28-CRP<2.5 (n = 62) | SDAI≦3.3 (n = 90) | DAS28-MCP-1<2.2 (n = 80) |
|---|---|---|---|---|
| Breg>39.22% | 87/99 = 87.88% | 51/62 = 82.26% | 77/90 = 85.56% | 69/80 = 86.25% |
| Tr1>2.64% | 92/99 = 92.93% | 57/62 = 91/94% | 85/90 = 94.44% | 76/80 = 95.00% |
| M2>2000/ml | 55/99 = 55.56% | 35/62 = 56.45% | 47/90 = 52.22% | 39/80 = 48.75% |
| IL-4≧31.3* | 18/99 = 18.18% | 12/62 = 19.35% | 37/90 = 41.11% | 15/80 = 18.75% |
| IL-5≧3.9 | 11/99 = 11.11% | 6/62 = 9.68% | 8/90 = 8.89% | 8/80 = 10.00% |
| IL-9≧0 | 99/99 = 100% | 62/62 = 100.00% | 90/90 = 100.00% | 80/80 = 100.00% |
| IL-10≧7.8 | 42/99 = 42.42% | 24/62 = 38.71% | 39/90 = 43.33% | 39/80 = 48.75% |
| IL-13≧0 | 99/99 = 100.00% | 62/62 = 100.00% | 90/90 = 100.00% | 80/80 = 100.00% |
| IL-27≧975.16 | 99/99 = 100.00% | 62/62 = 100.00% | 89/90 = 98.89% | 80/80 = 100.00% |
| IL-35≧0 | 99/99 = 100.00% | 62/62 = 100.00% | 90/90 = 100.00% | 80/80 = 100.00% |
| TGF-beta1≧903 | 0/98 = 0.00% | 0/61 = 0.00% | 0/89 = 0.00% | 0/79 = 0.00% |
| TNF-R1≧484 | 41/99 = 41.41% | 26/62 = 41.94% | 36/90 = 40.00% | 35/80 = 43.75% |
| TNF-R2≧829 | 22/99 = 22.22% | 12/62 = 19.35% | 20/90 = 22.22% | 21/80 = 26.25% |

Breg: B regulatory cells; Trl: T regulatory type 1 cells; M2: M2 macrophages, alternatively activated macrophage. All cytokines are expressed as pg/mL. Shown are positive visits with cells or cytokines in normal range or higher divided by all visits in different DAS28-score-based remission statuses to obtain individual percentages (%). All percentages in each item for different DAS28-score-based remission statuses compared with that for DAS28-ESR remission were non-significant by odds ratio (all *P*-values > 0.05). *The exceptions were for IL-4≧31.3 pg/mL: SDAI vs. DAS28-ESR gave an odds ratio of 2.261 and *P*-value = 0.01; SDAI vs. DAS28-CRP provided an odds ratio of 2.124 and *P*-value = 0.04; SDAI vs. DAS28-MCP-1 offered an odds ratio 2.193 and *P*-value = 0.02.

**Supplementay Table S4.** Comparison of immunoregulatory cells and cytokines between anti-CCP-positive (n = 35) and -negative (n= 15) subgroups in RA patients at baseline

| Items | *P*-values |
| --- | --- |
| Breg | 0.970* |
| Tr1 | 0.612 |
| M2 | 0.738 |
| IL-4 | 0.744 |
| IL-5 | 0.949 |
| IL-9 | 0.445 |
| IL-10 | 0.721 |
| IL-13 | 0.680 |
| IL-27 | 0.718 |
| IL-35 | 0.093 |
| TGF-β1 | 0.489 |
| sTNF-R1 | 0.411 |
| sTNF-R2 | 0.374 |

Breg: regulatory B cells; Tr1: T regulatory type 1 cells; M2: alternately activated macrophages, M2 macrophages. *Compared by t-test; all others were compared by Mann-Whitney U test.

**Supplementary Table S5.** Comparison of the percentage/number of immune-regulatory cells and cytokine levels between low and high ultrasound grades[#]

| | **Grade ≦ 1 (n = 89)** | **Grade > 1 (n = 58)** | ***P*-values** |
|---|---|---|---|
| Breg | 51.22 ± 14.03 | 53.74 ± 13.24 | 0.279* |
| Tr1 | 12.96 ± 9.20 | 14.02 ± 9.51 | 0.472 |
| M2 | 3262.92 ± 2476.77 | 5029.31 ± 7301.57 | 0.821 |
| IL-4 | 21.72 ± 45.88 | 32.62 ± 77.33 | 0.711 |
| IL-5 | 7.36 ± 29.41 | 12.19 ± 44.78 | 0.106 |
| IL-9 | 49.32 ± 73.21 | 58.11 ± 92.18 | 0.860 |
| IL-10 | 147.00 ± 684.85 | 284.97 ± 1169.27 | 0.635 |
| IL-13 | 446.71 ± 624.95 | 561.45 ± 1069.92 | 0.818 |
| IL-27 | 3568.55 ± 3659.51 | 3883.66 ± 3849.41 | 0.339 |
| IL-35 | 947.92 ± 1535.33 | 748.75 ± 1374.52 | 0.630 |
| TGF-beta1 | 33.81 ± 25.92 | 44.53 ± 34.35 | 0.071 |
| TNF-R1 | 480.96 ± 178.52 | 494.55 ± 214.66 | 0.367 |
| TNF-R2 | 601.51 ± 359.17 | 668.04 ± 426.43 | 0.593 |

[#]Ultrasound scoring system: see ref. 20. Combined ultrasound groups: Grade ≤ 1, comprising grade 0 and grade 1; grade > 1, comprising grade 2 and grade 3. Breg: B regulatory cells; Trl: T regulatory type 1 cells; M2: alternatively activated macrophage, M2 macrophages. All cytokines are expressed as pg/mL. *Compared by t-test; other comparisons were done by Mann-Whitney U test. All *P*-values > 0.05.

**Supplementary Table S6.** The correlation between DAS28-MCP-1 scores and other Disease Activity scores

| With which DAS28-MCP-1 correlates | Correlation coefficients | *P*-values |
|---|---|---|
| DAS28-ESR (all visits = 147) | 0.727 | <0.001 |
| With ESR≧28 mm/hr (n = 22) | 0.854 | <0.001 |
| With ESR<28 mm/hr (n = 125) | 0.786 | <0.001 |
| DAS28-CRP (all visits = 147) | 0.797 | <0.001 |
| With CRP≧10 mg/L (n = 13) | 0.783 | 0.003 |
| With CRP<10 mg/L (n = 134) | 0.837 | <0.001 |
| SDAI (all visits = 147) | 0.859 | <0.001 |
| With CRP≧10 mg/L (n = 13) | 0.783 | 0.003 |
| With CRP<10 mg/L (n = 134) | 0.875 | <0.001 |

DAS28: Disease Activity Score28; SDAI: Simplified Disease Activity Index. All correlations were done by Spearman's correlation method.

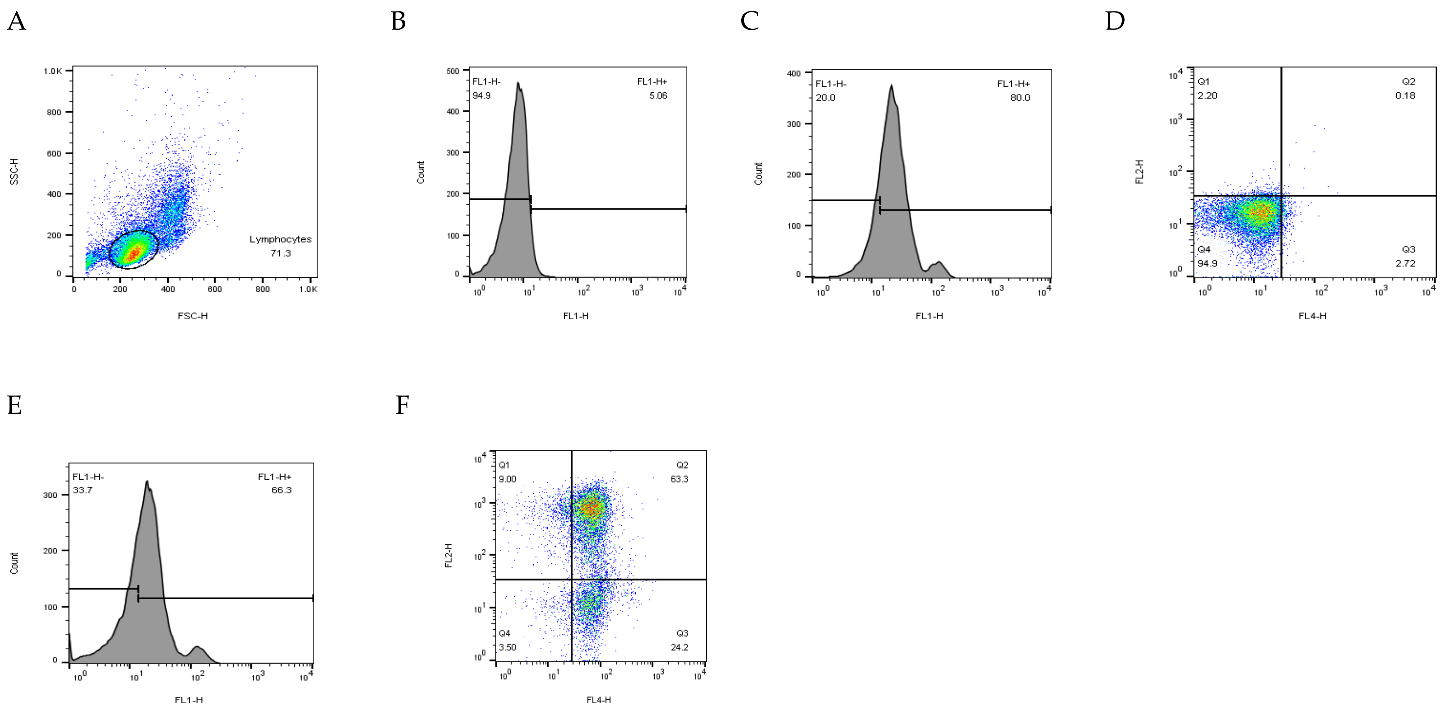


Supplementary Figure S1. The staining procedure for Breg cells. (A) Lymphocytes gated from peripheral blood mononuclear cells. (B) Mouse IgG1k-FITC as an isotype control and a background staining of lymphocytes. (C) Attain CD19+ B cells by CD19-FITC staining. (D) Mouse IgG1k-PE and IgG1k-APC as isotype controls and background staining for $CD19^{+}$ B cells. (E) Set CD5-PE and CD1d-APC double staining. (F) A bivariate plot to obtain $CD5^{+}$-PE and $CD1d^{high}$-APC Breg cells (already $CD19^{+}$): the upper right quadrant (Q2).

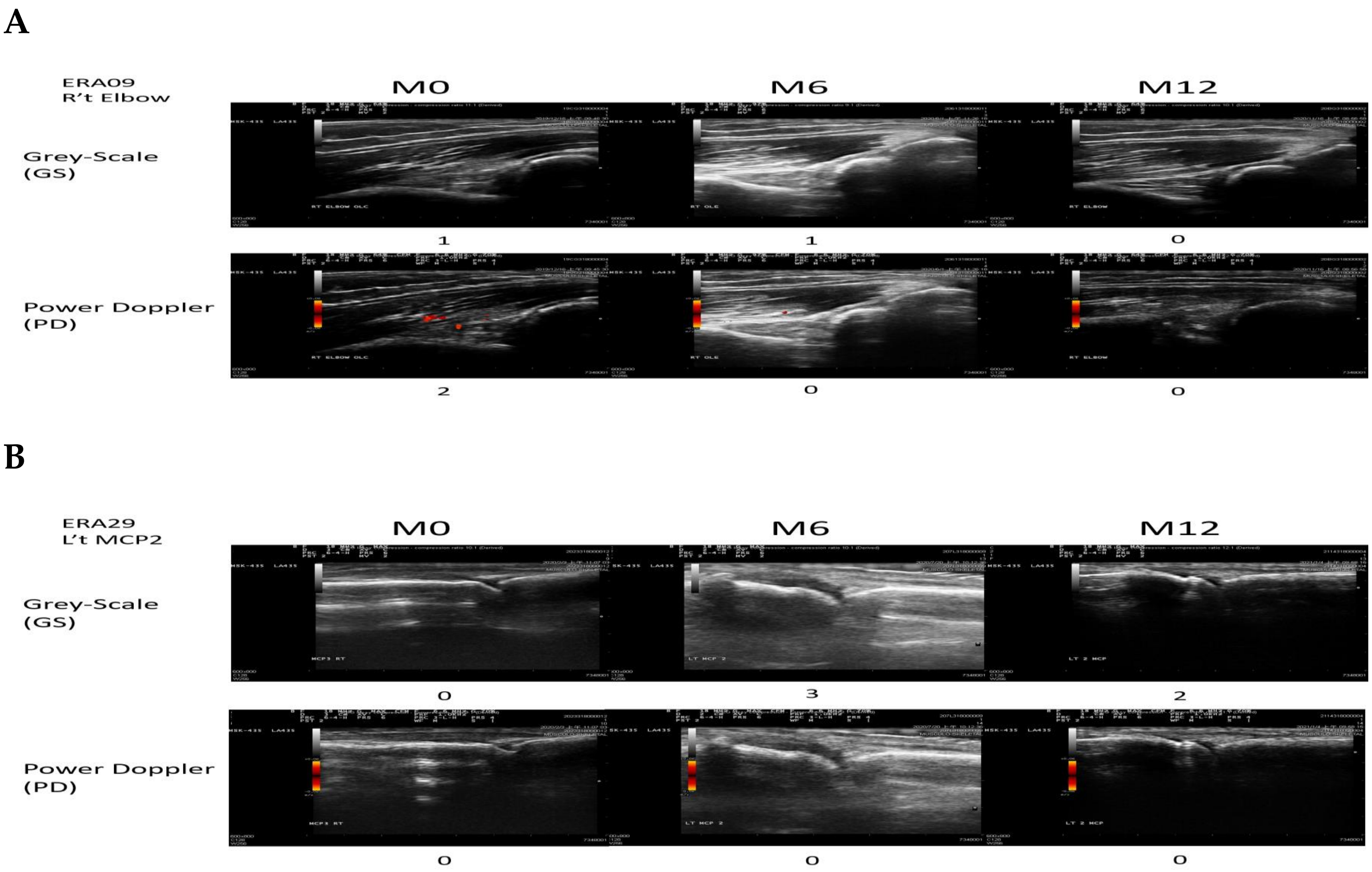
A
ERA09
R't Elbow
M0
M6
M12
Grey-Scale
(GS)
1
1
0
Power Doppler
(PD)
2
0
0
B
ERA29
L't MCP2
M0
M6
M12
Grey-Scale
(GS)
0
3
2
Power Doppler
(PD)
0
0
0

**Supplementary Figure S2.** Ultrasound grades readings from: (A) The right elbow joint of a patient. The numbers below each picture designated the grade readings based on scoring standards described in ref. 20. (B) The left 2$^{nd}$ MCP joint of another patient. The numbers below each picture designated the grade readings based on scoring standards described in ref. 20. M0, M6, and M12 indicate Month 0 (baseline), Month 6, and Month 12, respectively.
